# Using an Arabic Version of the Life-Space Assessment to Evaluate How Gait Speed and Gender Predict Mobility Restrictions among Older Adults

**DOI:** 10.3390/medicina60030411

**Published:** 2024-02-28

**Authors:** Alia A. Alghwiri, Faten S. Obeidat, Joud Al-Jaghbeer, Reham A. Abuatiq, Susan L. Whitney

**Affiliations:** 1 Department of Physiotherapy, School of Rehabilitation Sciences, The University of Jordan, Amman 11942, Jordan; 2 Department of Physical Therapy, School of Health and Rehabilitation Sciences, University of Pittsburgh, Pittsburgh, PA 15260, USA; whitney@pitt.edu; 3 Department of Hearing and Speech Sciences, School of Rehabilitation Sciences, The University of Jordan, Amman 11942, Jordan; faten.obeidat@ju.edu.jo; 4 Department of Physiotherapy, Faculty of Allied Medical Sciences, Middle East University, Amman 11831, Jordan; joudjaghbeer@gmail.com; 5 Rehabilitation Medicine Department, University of Washington, Seattle, WA 98195, USA; rabuat@uw.edu; 6 Department of Otolaryngology, School of Medicine, University of Pittsburgh, Pittsburgh, PA 15260, USA

**Keywords:** Arabic, gait, fear of falling, participation, restrictions, reliability, validity

## Abstract

*Background and Objectives*: The Life-Space Assessment (LSA) serves as an assessment tool for evaluating mobility and participation in older adults. To date, no studies have investigated the validity and reliability of the LSA within Arabic-speaking communities. The purpose of this study was to examine the reliability and validity of an Arabic version of the LSA and to investigate the potential predictors of mobility restrictions in older Arabic-speaking adults. *Materials and Methods*: This study involved a cohort of 75 Arabic-speaking older adults (with a mean age of 67.2 ± 5.9). The LSA was administered twice, with a one-week interval, to assess its test–retest reliability. The internal consistency and test–retest reliability of the LSA were assessed using Cronbach’s alpha and intra-class correlation coefficients (ICCs), respectively. The validity of the LSA was determined by analyzing its correlation with outcome measures related to the fear of falling, depression, quality of life, lower limb strength, physical performance, and gait speed. *Results*: The test–retest reliability of the LSA composite score demonstrated good results (ICC = 0.83). The validity of the LSA was supported by significant correlations between its scores and factors such as gender, education level, and all other outcome measures. Notably, being female and having a lower gait speed emerged as significant predictors of mobility restrictions in older Arabic-speaking adults, accounting for 49% of the variance (R^2^ = 49%) in the multiple logistic regression analysis conducted. *Conclusions*: The Arabic version of the LSA has proven to be a reliable and valid measure of mobility and participation among older Arabic-speaking adults. This study endorses the application of the Arabic LSA in both research and clinical settings involving older adults and emphasizes the need for further investigation to fully understand its psychometric features in other Arabic-speaking individuals afflicted with neurological and musculoskeletal conditions.

## 1. Introduction

Aging is a global phenomenon that is expected to have significant implications for society, economies, and healthcare sectors [1]. It is expected that the increase in the percentage of older adults will continue to rise in the future. Based on the World Health Organization’s (WHO) statistics, it is estimated that between 2020 and 2050, the proportion of persons who are 60 years or older will double [2], emphasizing the urgency for comprehensive strategies to address the challenges and harness the opportunities associated with this demographic transformation.

One of the key drivers of the aging population is the remarkable progress made in healthcare. Several factors have contributed to the aging population, including advances in healthcare and decreased mortality rates, which lead to increased life expectancy [3]. Advances in medical science, disease prevention, and treatment modalities have contributed significantly to increased life expectancy. As individuals benefit from improved healthcare, they not only survive into older age but also experience an enhanced quality of life. Concurrently, reduced mortality rates have become a hallmark of contemporary healthcare systems, allowing a larger proportion of the population to enter the ranks of the elderly. The intertwining effects of medical breakthroughs and decreased mortality contribute to the demographic shift, creating a world where a growing number of people are living longer than ever before.

Globally, there is a demographic change taking place as people live longer. This global demographic change is not limited to the developed world [3], but it is also happening in developing countries [4]. Similar demographic changes are taking place throughout the Arab world, where the population of the older adults is steadily growing. The United Nations classified Arab countries into fast-, moderate-, and low-aging categories, with Jordan being in the moderate-aging category [4].

As a result of increased life expectancy worldwide, non-communicable diseases and disabilities are becoming more common as populations age. Therefore, a significant demand for healthcare services is anticipated, since the aging process has both physical and mental consequences [5]. As individuals live longer lives, they are more likely to contend with a myriad of health challenges, ranging from chronic conditions to limitations in physical and mental functions.

One of the primary consequences of aging that causes disability in older persons living in the community is mobility restrictions [5]. Maintaining physical mobility is a crucial aspect of independent living and plays a significant role in determining the quality of life (QOL) for older adults [6]. Yet, many older persons suffer from mobility restrictions that affect their ability to perform daily living activities and participate actively in their communities [7].

Mobility restrictions in older adults can negatively impact their well-being, leading to reduced autonomy and social interactions, as well as an increased risk of isolation. This can also contribute to chronic conditions like cardiovascular diseases, obesity, and musculoskeletal problems. Addressing these challenges requires a comprehensive approach that includes preventive measures, rehabilitative interventions, and community support systems. Healthcare providers, policymakers, and communities must collaborate to develop strategies that promote active aging, ensuring older individuals maintain their physical independence and participate in their communities. Recognizing the importance of addressing mobility restrictions can foster a conducive environment for older adults, promoting their health and well-being.

Mobility restrictions were reported in 26.9% of older adults in the United States [8]. Personal factors and other environmental barriers may influence mobility restrictions in older adults [7,9]. Mobility restrictions are a major health concern for older adults and can have significant impacts on their physical and mental health [10,11], which may raise mortality risks [12]. Therefore, it is essential to use valid instruments to measure mobility in older adults. One of the widely used tools to assess mobility restrictions in older adults is the Life-Space Assessment (LSA) [13].

The LSA is a self-reported questionnaire that was developed to quantify mobility in older adults [13]. It asks about the distance an individual travels to perform daily living activities and quantifies how far and how often the individual moves out of their place of residence and the degree of independence in performing those activities. The LSA is a valid and reliable tool that has been used extensively in older adults [13,14] and other populations [15,16]. The LSA has been translated into several languages and adapted to various cultures, such as German [17] and Chinese [18]. However, no studies have investigated the validity and reliability of the LSA within Arabic-speaking communities.

The lack of an Arabic version of the LSA limits its use in the Arabic-speaking countries and necessitates an adaption of this measure into the Arabic language. Therefore, the aims of this study were to translate the LSA into the Arabic language, examine its psychometric properties in a sample of older Arabic-speaking adults, and investigate the potential predictors of mobility restrictions in older Arabic-speaking adults.

## 2. Materials and Methods

A reliability study was utilized to translate the LSA into the Arabic language, validate it with other outcome measures, and culturally adapt it into the Arabic nation under study. Ethics approval for the conduction of this study was obtained from the institutional review board of Jordan University Hospital (2021/134).

### 2.1. Participants

Community-dwelling individuals who were 60 years or older (according to the United Nations report) [3] from all genders were included. The exclusion criterion was having any serious comorbidities that affected the individual’s mobility, such as neurological or orthopedic disorders. However, individuals with common chronic conditions such as diabetes and hypertension that did not affect their general health were included. Older adults were invited to participate in this study between January and July 2023 via paper and social media advertisements.

### 2.2. Translation and Validation Processes

The LSA was translated based on international guidelines and procedures [19]. The translation and validation of the LSA into Arabic involved several steps. A bilingual therapist translated the LSA from English into the Arabic language. A review of the translated version was conducted by a panel of experts in the field to ensure accuracy and clarity. Afterward, a backward translation of the Arabic version of the LSA was performed into English. The panel of experts compared the backward translated version with the original English version and made final edits to the final Arabic version of the LSA to ensure that it was linguistically and culturally appropriate.

The psychometric properties of the Arabic LSA, including reliability and validity, were evaluated. The reliability of the translated version was assessed by examining the internal consistency and test–retest reproducibility of the component and composite scores. The convergent validity of the Arabic LSA was assessed by comparing its composite score with other outcome measures related to the fear of falling (FOF), depression, QOL, lower limb strength, physical performance, and gait speed. The previously mentioned areas were selected for convergent validity based on their relationship to mobility and participation in the literature. Mobility and participation are multifaceted, encompassing demographics, health-related, psychosocial, and environmental factors. However, it is difficult to include all of these factors in one study. Therefore, we tried to include the main factors from each category in our study.

### 2.3. Procedures

Eligible participants were approached about participating in this study via advertisements in university schools, the university hospital, and social media. The primary investigator explained the details of the study to interested individuals and answered their questions and concerns before providing the consent form. After they agreed to participate in the study, they signed the consent form. Afterward, demographic information was collected, including age, gender, education level, marital status, height, and weight. Then, all older adults were provided with the study questionnaires and asked to read them carefully and answer the questions to the best of their knowledge. Study questionnaires included the Arabic LSA for the assessment of mobility, the Falls Efficacy Scale—International (FES-I) to quantify their FOF [20], the Medical Outcomes Study Short Form 12 (SF-12) to estimate their health-related QOL [21], and the Geriatric Depression Scale (GDS) for the screening of depression, if present [22], Finally, well-trained physical therapists conducted the physical-performance-based measurements for all participants, including the Short Physical Performance Battery (SPPB) for physical performance [23], knee extension strength for the evaluation of lower extremity strength, and gait speed assessment using the 4-Meter Walk Test [24]. One week later, a random sample of the participants was approached again to complete the Arabic LSA to examine its test–retest reliability.

### 2.4. Outcome Measures

*Life-Space Assessment*

The LSA is a self-reported measure that identifies mobility restrictions in individuals during the previous four weeks [13]. The LSA has 5 levels: “level 1 mobility” is restricted to other rooms of one’s home besides their sleeping room; “level 2 mobility” is restricted to areas outside of one’s home (such as the porch, garage, or driveway); “level 3 mobility” is limited to one’s neighborhood other than one’s yard or apartment building; “level 4 mobility” is within one’s town; and “level 5 mobility” is outside of one’s town [13]. The composite score of the LSA ranges between 0 and 120. A higher score indicates better community mobility skills and social participation [13]. A cut-off score of 78.5 was reported for individuals with spinal cord injuries who were mobile in their communities [25]. The LSA has demonstrated very good psychometric properties in older adults [26].

*Medical Outcomes Study Short Form 12 (SF-12)*

SF-12 is a 12-item questionnaire that examines health-related QOL [21]. SF-12 includes the physical and mental aspects of QOL and creates subscale scores [21]. The total score of the SF-12 ranges from 0 to 100, with a higher score indicating a better QOL [27]. According to Jakobsson (2007), the SF-12 is a valid and reliable test for assessing QOL in older adults [28]. The Arabic version of SF-12 had very good psychometric properties [29].

*Gait Speed (4-Meter Walk Test)*

Gait speed is an important performance-based measure of mobility that provides essential information about individual’s physical functioning and overall health [30]. Several forms of gait speed are available to use; however, the 4-Meter Walk Test was the most widely used gait speed test in older adults based on a recent systematic review [24]. The time it takes a person to walk 4 m at their normal speed is recorded. The participant begins the test from a standing position and walks towards a specified endpoint in a straight line.

*Falls Efficacy Scale—International (FES-I)*

The FES-I was developed to assess FOF in older adults by measuring an individual’s confidence in performing a range of activities of daily living without losing balance [20]. The FES-I asks about 16 activities that can be scored using a 4-point Likert scale (1 = not at all concerned, 2 = somewhat concerned, 3 = fairly concerned, and 4 = very concerned) [31]. The total score ranges from 16 to 64, with a higher score indicating a greater FOF. The Arabic version of the FES-I demonstrated good validity and reliability for use for Arabic-speaking individuals [32].

*Geriatric Depression Scale*

The short Geriatric Depression Scale (GDS-15) is used to screen for symptoms of depression in older adults [22]. The scale consists of 15 items that can be answered with either “Yes” or “No”, which creates a total score that ranges from 0 to 15. The Arabic version of GDS-15 showed good psychometric properties [33].

*Short Physical Performance Battery (SPPB)*

The SPPB is a performance-based test that was developed to quantify general physical performance in older adults [23]. It consists of five components: feet together for 10 s, a semi-tandem stand for 10 s, tandem standing for 10 s, a four-meter timed walk, and the time needed for five chair-stands. A score of 8 or less out of 12 is considered a poor functional performance [23]. The SPPB was reported to have good validity and reliability [34].

*Knee extension strength using a dynamometer*

Knee extension strength was tested using an electrical handheld dynamometer (Lafayette Instrument, Model 01163). Participants were instructed to sit on a highchair with their feet off the ground and their knees flexed at 90 degrees. The dynamometer was secured with a belt to test the strength of the quadriceps. The test was repeated twice, and the average score was reported [35]. This test has good concurrent validity compared with the gold standard (isokinetic dynamometer) [35].

### 2.5. Statistical Analysis

Descriptive and inferential statistics were used to describe demographics and health-related information. The internal consistency of the LSA items were calculated using Cronbach’s alpha. The test–retest reliability of the items and total score of the Arabic LSA was examined using the intraclass correlation coefficient (ICC) and 95% confidence interval (CI). Furthermore, the Spearman correlation coefficient was used to calculate the association between the Arabic LSA and other study variables to assess its validity. Logistic regression was used with the LSA cut-off score to explore potential factors that may predict mobility restrictions. We started with simple logistic regression for each factor. Then, all factors with *p* < 0.25 were added to the multiple logistic regression analysis to identify the significant predictors among them. The Statistical Package for the Social Sciences (SPSS), Version 28.0 (SPSS Inc., Chicago, IL, USA) was used for the analysis. A *p*-value of 0.05 or less was set as statistically significant.

## 3. Results

Seventy-five community-dwelling older adults participated in this study, with 62.7% females and a mean ± SD age of 67.2 ± 5.9 years. The sample characteristics and the mean ± SD of the outcome measure results are presented in Table 1. Based on the LSA cut-off score, 61.3% of our sample were found to be mobility-dependent.

Cronbach’s alpha was found to be 0.81, which indicates a good level of internal consistency for the Arabic LSA in older adults. The test–retest reliability of the Arabic LSA item and composite scores ranges between 0.57 for the first level (bedroom) and 0.84 for the third level (outside the house) (Table 2). The Arabic LSA had significant correlations with FES-I (r = 0.67), female gender (r = 0.60), quadricep strength (r = 0.56), gait speed (r = 0.48), GDS (r = 0.47), SF-12 (r = 0.47), SPPB (r = 0.34), and education level (r = 0.30) (Table 3).

Using simple logistic regression, all the factors were eligible for entry into multiple logistic regression analysis. However, after conducting multiple logistic regression analysis, two main factors were associated with an increased likelihood of having mobility restrictions in older Arabic-speaking adults (R^2^ = 49%): female gender and slower gait speed (Table 4).

## 4. Discussion

The aims of this study were to translate the LSA into the Arabic language, examine its psychometric properties in a sample of older Arabic-speaking adults, and investigate the potential predictors of mobility restrictions in older Arabic-speaking adults. The good internal consistency and good test–retest reliability, along with the significant correlations with the study measures, suggests that the Arabic LSA is a valid and reliable tool for evaluating mobility and independence in older Arabic-speaking adults. Based on the LSA cut-off score, 61.3% of our sample had mobility restrictions.

The translation and validation of the LSA into Arabic provides healthcare professionals with an assessment tool to evaluate mobility in Arabic-speaking communities, which may assist with identifying mobility restrictions and create focused interventions to enhance mobility and QOL. Moreover, the use of a valid and reliable instrument in the patients’ first language can aid in enhancing communication between healthcare providers and Arabic-speaking patients, which might improve health outcomes [36]. The availability of the Arabic LSA can encourage intercultural research and collaboration, which could increase our understanding of aging and mobility in different nations.

One important aspect of the LSA is its test–retest reliability, which assesses the consistency of scores over time without a real change in an individual’s status. The test–retest reliability of the Arabic LSA composite score in our study was 0.83, which is comparable to the findings of other studies [13,25,37]. A test–retest reliability score of 0.86 was reported in the original English LSA over a two-week interval [13]. The Swedish LSA version also had a test–retest reliability between 0.84 and 0.94 over a two-week period [37]. In our study, the other LSA items had lower test–retest reliability scores than the composite score. These findings can be explained by the fluctuations that older adults may experience in their mobility levels due to various factors, such as health conditions, the weather, social activities, or personal preferences [38]. Specifically, the weather may have influenced our sample, since we conducted our study in both the winter and summer seasons. Older adults do not prefer to go outside the home during rainy or snowy weather, since they have a fear of falling. Another justification for our findings may be the recall bias, since the LSA asks older adults to recall their mobility patterns in the past week, which may be challenging for some individuals, especially those with memory problems. Older adults may also be influenced by social desirability, mood, or expectations when answering the LSA. They may over-report or under-report their mobility levels in one of the sessions to present themselves in a more favorable or realistic way or to match their perceived norms or goals. Overall, the LSA has good test–retest reliability over short-term intervals in older community-dwelling adults, which indicates that it can be used as a consistent and reliable tool to measure mobility and participation in older adults over time [13].

Mobility restrictions are a major health concern for older adults, and they are multifactorial; identifying factors that are associated with mobility and participation is crucial. Several studies examined the association between the LSA and other factors in older adults [14,18,39]. Curcio et al. found that mobility restrictions were significantly associated with lower education and income, depressive symptoms, and cognitive impairment, whereas a higher LSA score was associated with better SPPB performance [14]. Ullrich et al. found significant relationship between the LSA and outdoor physical activities, FOF, and motor status [39]. Tseng et al. also found a significant correlation between the LSA and depressive symptoms and QOL in older Chinese adults [18].

In our study, the Arabic LSA had significant correlations with female gender, gait speed, FOF, quadricep strength, general physical performance, depression symptoms, education level, and QOL. Older adults with higher FOF tend to restrict their mobility due to a perceived risk of falling. As a result, they may have lower LSA scores, reflecting limited life-space mobility. The correlation between LSA and FOF in our study (−0.67) demonstrated an inverse significant relationship, emphasizing that older participants with FOF concerns had lower mobility scores. Quadricep strength also plays a crucial role in functional mobility. Strong quadricep muscles enable better walking ability, stair climbing, and overall movement. Older adults with stronger quadriceps are likely to have higher LSA scores due to improved physical performance. Similarly, better general physical performance, including strength, endurance, and balance, contributes to higher LSA scores. Psychological aspects, especially depression, can significantly impact an individual’s motivation, energy levels, and social engagement. Older adults experiencing depressive symptoms may withdraw from social interactions and limit their life-space interactions. Consequently, LSA scores may be lower in this group. Education influences lifestyle choices, access to resources, and social participation. Higher education levels are associated with broader life-space mobility, as educated individuals often engage in diverse activities and have a better awareness of community resources. Life-space mobility has a direct relationship to participants’ QOL (0.47). Older adults with broader life-space experiences report better QOL. Positive correlations between LSA scores and QOL measures highlight the importance of maintaining mobility for overall well-being. In summary, the LSA’s significant correlations with these factors underscore its validity as a measure of life-space mobility in older adults. Researchers and clinicians can use the LSA to assess and monitor mobility limitations, identify risk factors, and tailor interventions to enhance overall well-being.

Identifying factors that predict mobility restrictions in older Arabic-speaking adults has several benefits. First, it can help healthcare providers identify older Arabic adults who are at risk for mobility restrictions and develop interventions to prevent and manage these restrictions. Second, identifying factors that contribute to mobility restrictions can help healthcare providers develop targeted interventions to address these factors and improve mobility in older adults.

Female gender and slower gait speed were found to be significant predictors of mobility restrictions in older Arabic-speaking adults, explaining 49% of the variance. The female gender is one of the factors that has been well investigated in older adults. In our study, being an older female was found to be a significant predictor for mobility restrictions (−0.60). Ullrich et al. reported the same finding as ours and suggested a “gender-specific approach” to improve older female adults’ mobility [39]. Curcio et al. also found that females in Columbia and Brazil were more likely to exhibit lower LSA scores than males [14]. Gait speed is another important health variable. A recent study (2020) demonstrated a well-established relationship between gait speed and mobility restrictions in older adults, which is in line with our findings [40]. Gait speed has been related to long-term survival in older persons [30]. A faster gait speed is associated with greater independence in daily activities. Older adults with faster gait speeds cover larger life-space areas, resulting in higher LSA scores. Conversely, a slower gait speed may limit life-space mobility.

## 5. Limitations

Overall, reliability studies can provide valuable information about the consistency of measurement tools but should be interpreted with caution. The translated version of the LSA requires further testing and validation to ensure its reliability and validity in other Arabic-speaking individuals with neurological and musculoskeletal disorders. Future research should further examine the psychometric properties of LSA in Arabic-speaking populations, particularly with regard to its sensitivity to change over time.

## 6. Conclusions

Mobility restrictions are prevalent among older adults, necessitating the use of reliable assessment tools. The LSA is a widely recognized tool for documenting and quantifying mobility among older adults. It has been employed in both clinical and research environments to assess the efficacy of interventions and forecast future health outcomes. The translation and validation of the LSA into Arabic is a crucial advancement in assessing mobility among Arabic-speaking populations. The Arabic version of the LSA holds promise for enhancing communication between healthcare professionals and Arabic-speaking patients, as well as promoting international research and collaboration. The identification of predictors of mobility restrictions in older adults is vital for developing effective strategies to prevent and manage these restrictions. Among Arabic-speaking seniors, gender (specifically being female) was found to be significantly associated with reduced mobility, while a faster gait speed was significantly linked to enhanced mobility.

## Figures and Tables

**Table 1:** Demographic information and clinical characteristics of the participants (N = 75).

Variable	Mean ± SD
Age	67.2 ± 5.9
Gender	
Male, n (%)	28 (37.3%)
Female, n (%)	47 (62.7%)
Marital status	
Single, n (%)	3 (4%)
Married, n (%)	57 (76%)
Widowed, n (%)	14 (18.7%)
Divorced, n (%)	1 (1.3%)
Years of education	13.7 ± 3.8
Smoking	
No, n (%)	58 (77.3%)
Yes, n (%)	17 (12.7%)
Number of medications	2.8 ± 1.9
FES-I	23.85 ± 5.1
GDS	1.57 ± 1.97
SF-12 Physical	68 ± 20.5
SF-12 Mental	74.6 ± 20
SF-12 Total	72 ± 17.5
SPPB	10 ± 1.5
Knee extension strength—right	25 ± 11.7
Knee extension strength—left	23 ± 11.1
Gait speed (meter/second)	1.3 ± 0.3
Life-Space Assessment total score	70.45 ± 21.3
Life-Space Assessment cut-off score	
Dependent ≤ 78, n (%)	46 (61.3%)
Independent ≥ 79, n (%)	29 (38.7%)

FES-I: Falls Efficacy Scale—International; GDS: Geriatric Depression Scale; SF-12: Medical Outcomes Study Short Form 12; SPPB: Short Physical Performance Battery.

**Table 2:** Test–retest reliability of the Life-Space Assessment (LSA) (N = 32).

Levels	ICC	95% CI	*p*-Value
(1) Bedroom	0.57	0.34–0.80	0.001
(2) Home	0.68	0.47–0.81	0.001
(3) Outside the house	0.84	0.67–0.93	0.001
(4) Neighborhood	0.71	0.32–0.87	0.001
(5) Town	0.76	0.51–0.88	0.001
LSA—total score	0.83	0.58–0.92	0.001

ICC: Intraclass correlation coefficient.

**Table 3:** Correlations between the Life-Space Assessment (LSA) and study variables (N = 75).

Variable	LSA (Spearman)
Age	0.03
Gender (female)	−0.60 **
Years of education	0.30 **
FES-I	−0.67 **
GDS	−0.47 **
SF-12 Physical	0.40 **
SF-12 Mental	0.42 **
SF-12 Total	0.47 **
SPPB	0.34 **
Knee extension strength—right	0.58 **
Knee extension strength—left	0.54 **
Gait speed	0.48 **

FES-I: Falls Efficacy Scale—International; GDS: Geriatric Depression Scale; SF-12: Medical Outcomes Study Short Form 12; SPPB: Short Physical Performance Battery. **: *p* < 0.001.

**Table 4:** Regression analysis of mobility restrictions in older adults.

Variables	Simple Logistic Regression		Multiple Logistic Regression	
	OR	*p*-Value	OR	*p*-Value
Age	1.047	0.243	1.056	0.488
Gender (female)	0.048	<0.001	0.046	0.001
Years of education	1.082	0.232	1.006	0.951
FES-I	0.708	<0.001	0.836	0.260
GDS	0.605	0.007	0.600	0.123
SF-12 Total	1.052	0.003	0.979	0.518
SPPB	1.750	0.004	1.237	0.537
Gait speed	13.272	0.004	20.250	0.040
Knee extension strength—right	1.130	0.001	0.982	0.862
Knee extension strength—left	1.150	0.001	1.040	0.673

FES-I: Falls Efficacy Scale—International; GDS: Geriatric Depression Scale; SF-12: Medical Outcomes Study Short Form 12; SPPB: Short Physical Performance Battery.

## Data Availability

The data that support the findings of this study are available from the corresponding author upon reasonable request.
